# Supplementary figures and images for: Bacterial diversity and composition on the rinds of specific melon cultivars and hybrids from across different growing regions in the United States
Source: PLoS One. 2024 Apr 11;19(4):e0293861. doi: 10.1371/journal.pone.0293861 (PMC11008840; doi:10.1371/journal.pone.0293861)

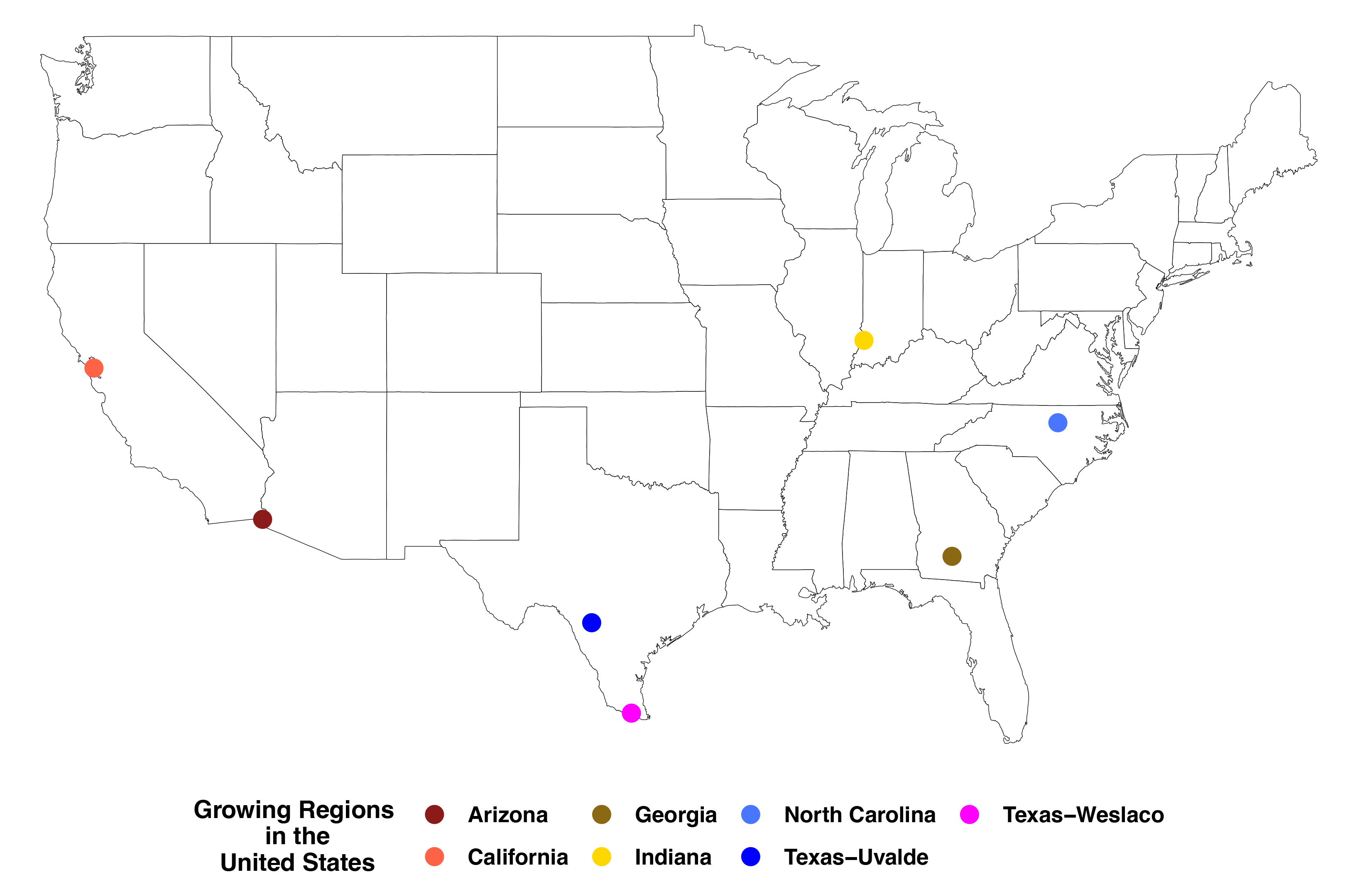

Supplement: S1 Fig — Map of the United States with location of each field that melons were grown and harvested during this study. Location marking of the field is based on the longitude and latitude of the field. Map was generated in R using the ggplot package version 3.4.1. (TIF) [file pone.0293861.s001.tif]

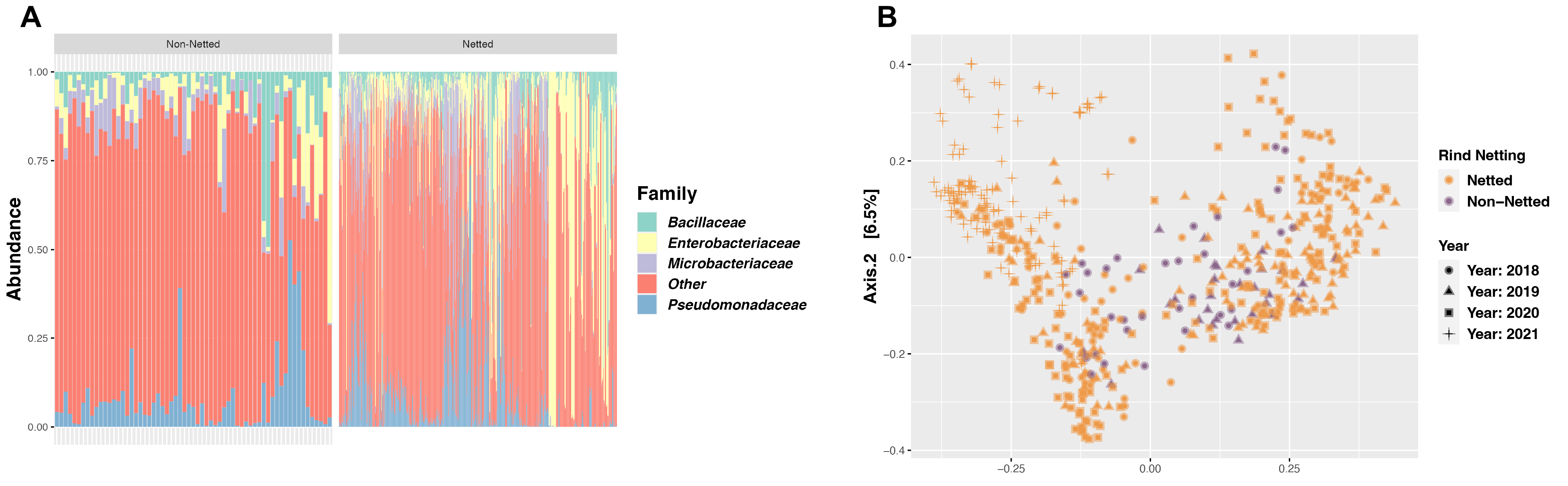

Supplement: S2 Fig — (A)Taxonomic relative abundance of netted and non-netted melons identified at the Family level. (B) Bray-Curtis PCoA plot clustered by the year and colored by the rind netting. (TIF) [file pone.0293861.s002.tif]

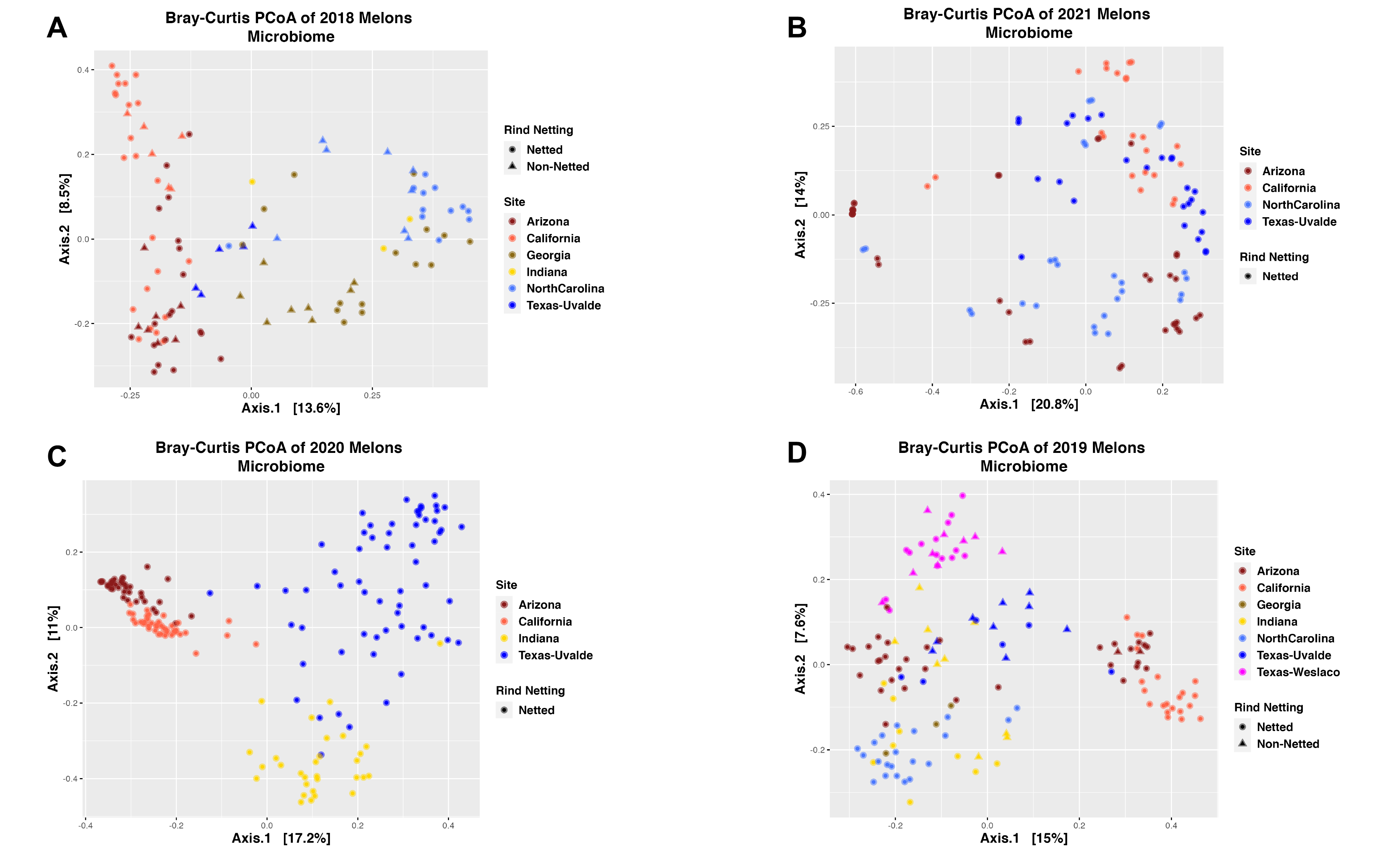

Supplement: S3 Fig — (A) Bray-Curtis PCoA plot clustered by the regions in 2018. (B) Bray-Curtis PCoA plot clustered by the regions in 2019. (C) Bray-Curtis PCoA plot clustered by the regions in 2020. (D) Bray-Curtis PCoA plot clustered by the regions in 2021. (TIF) [file pone.0293861.s003.tif]

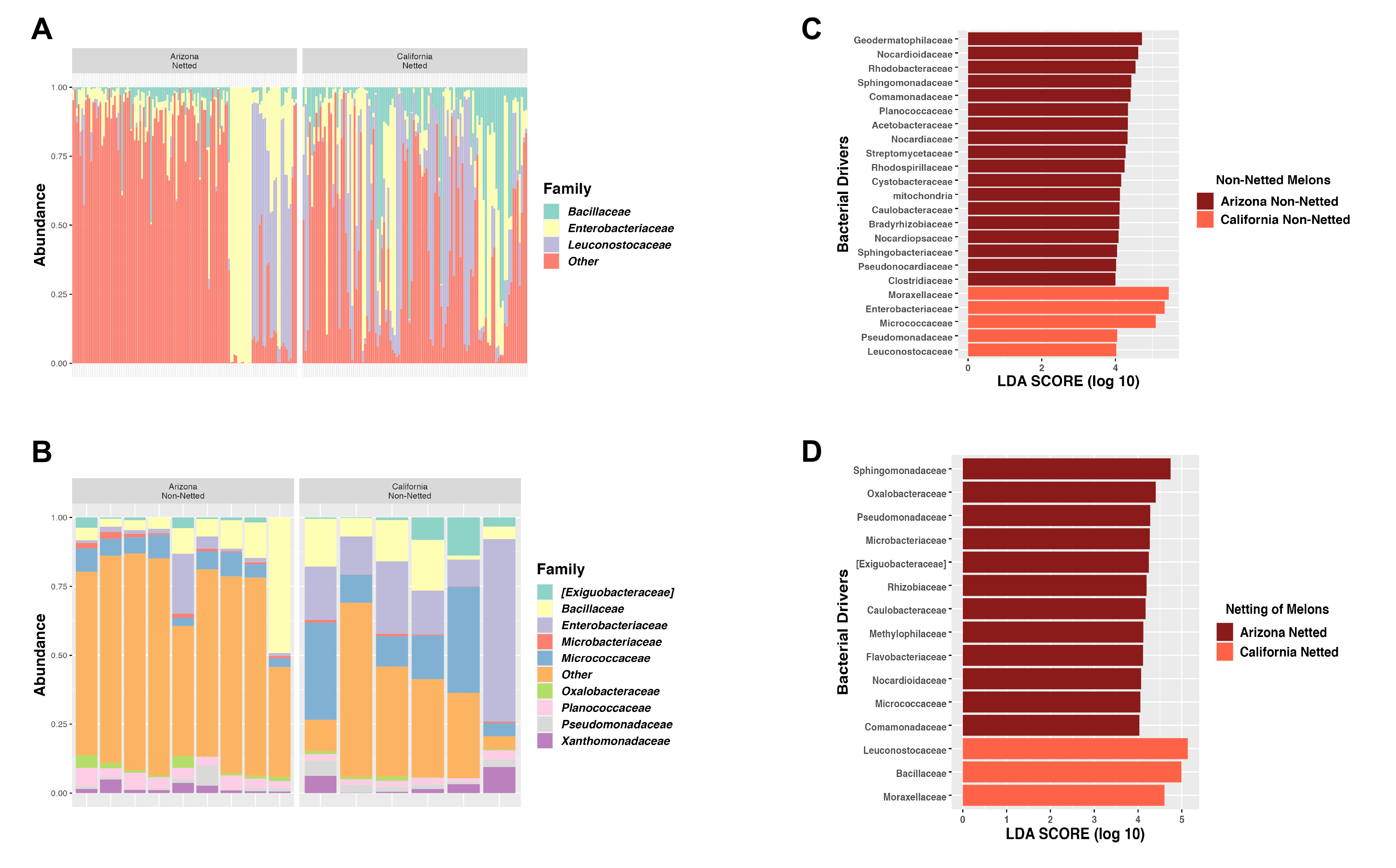

Supplement: S4 Fig — (A) Taxonomic relative abundance looking at Arizona and California for netted melons. (B) Taxonomic relative abundance looking at Arizona and California for non-netted melons. (C) Lefser analysis for Arizona and California netted melons. (D) Lefser analysis for Arizona and California non-netted melons (LDA = 4). (TIF) [file pone.0293861.s004.tif]

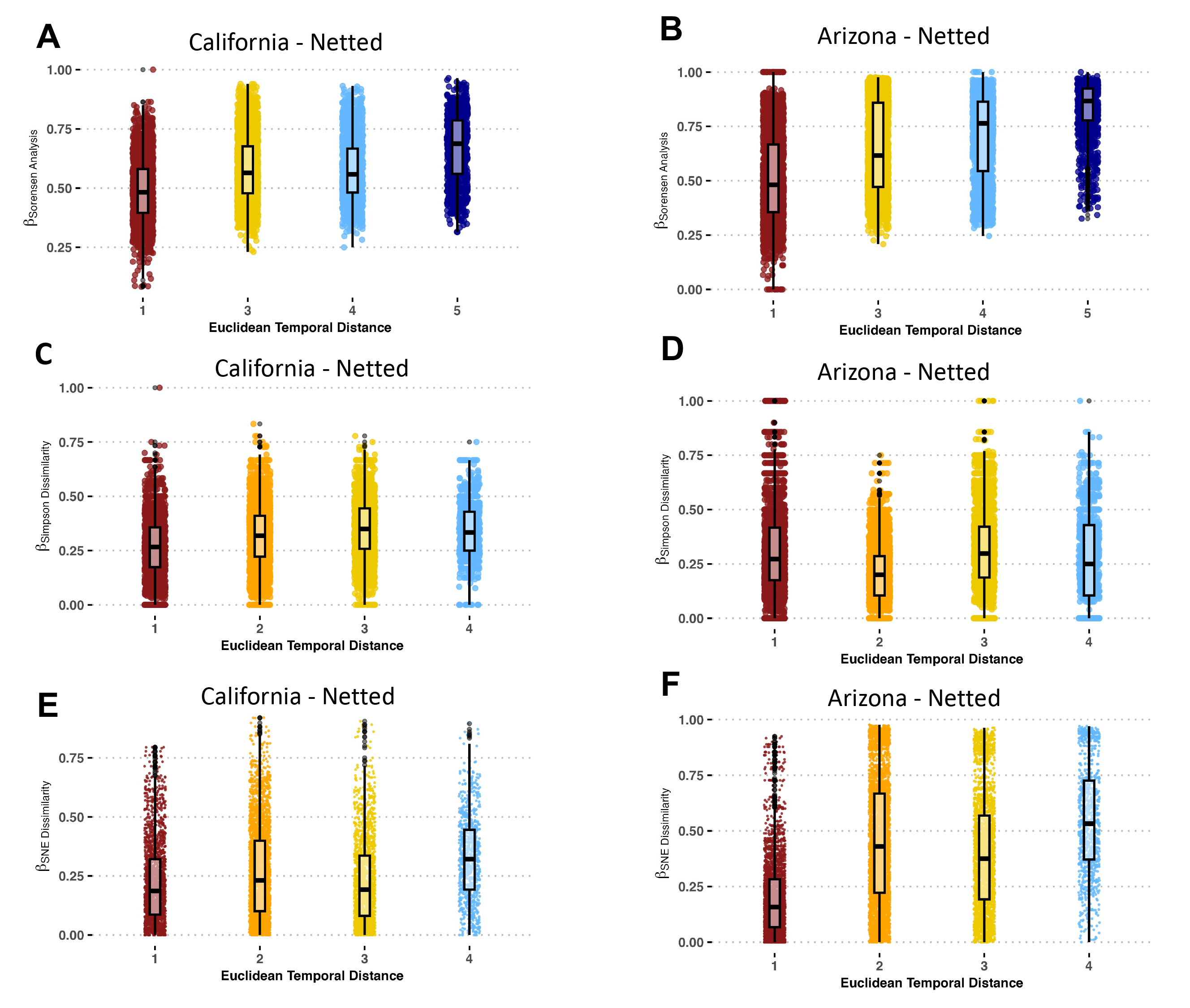

Supplement: S5 Fig — (A) β-Sorensen dissimilarity plotted against Euclidean temporal distance using mantel correlation to assess taxa presence or absence of bacterial communities for California netted melons. (B) β-Sorensen dissimilarity plotted against Euclidean temporal distance using mantel correlation to assess taxa presence or absence of bacterial communities for Arizona netted melons. (C) β-Simpson dissimilarity plotted against Euclidean temporal distance using mantel correlation to assess taxa replacement or turnover of bacterial communities for California netted melons. (D). β-Simpson dissimilarity plotted against Euclidean temporal distance using mantel correlation to assess taxa replacement or turnover of bacterial communities for Arizona netted melons. (E) β-SNE dissimilarity plotted against Euclidean temporal distance using mantel correlation to assess taxa nestedness of bacterial communities for California netted melons. (F) β-SNE dissimilarity plotted against Euclidean temporal distance using mantel correlation to assess taxa nestedness of bacterial communities for Arizona netted melons. (TIF) [file pone.0293861.s005.tif]
