# Supplementary material for: Bacterial diversity and composition on the rinds of specific melon cultivars and hybrids from across different growing regions in the United States
Source: PLoS One. 2024 Apr 11;19(4):e0293861. doi: 10.1371/journal.pone.0293861 (PMC11008840; doi:10.1371/journal.pone.0293861)
Supplement: S1 Table — (PDF) [file pone.0293861.s006.pdf]

S1 Table. Core bacterial families of netted and non-netted melons

Netting Type	Bacterial Family
Non-netted	*Pseudomonadaceae*<br>*Bacillaceae*<br>*[Exiguobacteraceae]*
Netted	None
