# Supplementary material for: Bacterial diversity and composition on the rinds of specific melon cultivars and hybrids from across different growing regions in the United States
Source: PLoS One. 2024 Apr 11;19(4):e0293861. doi: 10.1371/journal.pone.0293861 (PMC11008840; doi:10.1371/journal.pone.0293861)
Supplement: S2 Table — (PDF) [file pone.0293861.s007.pdf]

S2 Table. Core bacterial families of netted melons

Location	Bacterial Family	
Arizona	None	
California	*Leuconostocaceae*<br>*Bacillaceae*	
Texas-Uvalde	*Enterobacteriaceae*	
Texas-Weslaco	*[Exiguobacteraceae]*<br>*Bacillaceae*<br>*Caulobacteraceae*<br>*Enterobacteriaceae*<br>*Microbacteriaceae*<br>*Micrococcaceae*<br>*Moraxellaceae*<br>*Nocardioidaceae*	*Oxalobacteraceae*<br>*Paenibacillaceae*<br>*Planococcaceae*<br>*Pseudomonadaceae*<br>*Rhizobiaceae*<br>*Sphingomonadaceae*<br>*Streptomycetaceae*<br>*Xanthomonadaceae*
North Carolina	None	
Georgia	*[Exiguobacteraceae]*<br>*Enterobacteriaceae*<br>*Methylobacteriaceae*	*Microbacteriaceae*<br>*Pseudomonadaceae*<br>*Sphingomonadaceae*
Indiana	*Enterobacteriaceae*<br>*Methylobacteriaceae*<br>*Microbacteriaceae*<br>*Nocardiaceae*	*Nocardioidaceae*<br>*Pseudomonadaceae*<br>*Sphingomonadaceae*<br>*Xanthomonadaceae*
