# Supplementary material for: Bacterial diversity and composition on the rinds of specific melon cultivars and hybrids from across different growing regions in the United States
Source: PLoS One. 2024 Apr 11;19(4):e0293861. doi: 10.1371/journal.pone.0293861 (PMC11008840; doi:10.1371/journal.pone.0293861)
Supplement: S3 Table — (PDF) [file pone.0293861.s008.pdf]

S3 Table. Core bacterial families of non-netted melons

Location	Bacterial Family	
Arizona	*[Exiguobacteraceae]*<br>*Bacillaceae*<br>*Bradyrhizobiaceae*<br>*Comamonadaceae*<br>*Dermabacteraceae*<br>*Geodermatophilaceae*	*Nocardiaceae*<br>*Nocardioidaceae*<br>*Nocardiopsaceae*<br>*Planococcaceae*<br>*Pseudonocardiaceae*<br>*Rhodobacteraceae*
California	*[Exiguobacteraceae]*<br>*Bacillaceae*<br>*Enterobacteriaceae*<br>*Leuconostocaceae*<br>*Micrococcaceae*	*Moraxellaceae*<br>*Planococcaceae*<br>*Pseudomonadaceae*<br>*Xanthomonadaceae*
Texas-Uvalde	*[Exiguobacteraceae]*<br>*Enterobacteriaceae*<br>*Microbacteriaceae*<br>*Micrococcaceae*	*Moraxellaceae*<br>*Nocardioidaceae*<br>*Oxalobacteraceae*<br>*Pseudomonadaceae*
Texas-Weslaco	*[Exiguobacteraceae]*<br>*[Weeksellaceae]*<br>*Bacillaceae*<br>*Deinococcaceae*<br>*Enterobacteriaceae*<br>*Microbacteriaceae*<br>*Micrococcaceae*<br>*Moraxellaceae*	*Nocardioidaceae*<br>*Oxalobacteraceae*<br>*Planococcaceae*<br>*Pseudomonadaceae*<br>*Rhodobacteraceae*<br>*Sphingomonadaceae*<br>*Xanthomonadaceae*
North Carolina	*Bacillaceae*<br>*Enterobacteriaceae*<br>*Methylobacteriaceae*	*Microbacteriaceae*<br>*Pseudomonadaceae*<br>*Sphingomonadaceae*
Georgia	*Bacillaceae*<br>*Caulobacteraceae*<br>*Geodermatophilaceae*<br>*Methylobacteriaceae*	*Microbacteriaceae*<br>*Nocardiaceae*<br>*Sphingomonadaceae*
Indiana	*[Exiguobacteraceae]*<br>*Bacillaceae*<br>*Caulobacteraceae*<br>*Enterobacteriaceae*<br>*Methylobacteriaceae*<br>*Microbacteriaceae*	*Micrococcaceae*<br>*Moraxellaceae*<br>*Nocardiaceae*<br>*Nocardioidaceae*<br>*Oxalobacteraceae*
