# Supplementary material for: Bacterial diversity and composition on the rinds of specific melon cultivars and hybrids from across different growing regions in the United States
Source: PLoS One. 2024 Apr 11;19(4):e0293861. doi: 10.1371/journal.pone.0293861 (PMC11008840; doi:10.1371/journal.pone.0293861)
Supplement: S4 Table — (PDF) [file pone.0293861.s009.pdf]

S4 Table. Core bacterial families of Arizona and California melons

Melon Type	Bacterial Family	
Non-netted melons	*[Exiguobacteraceae]*<br>*Bacillaceae*	*Micrococcaceae*<br>*Planococcaceae*
Netted melons	None	
